# Inhibitory effect of high leucine concentration on α-amylase secretion by pancreatic acinar cells: possible key factor of proteasome

**DOI:** 10.1042/BSR20181455

**Published:** 2018-12-11

**Authors:** Long Guo, Baolong Liu, Chen Zheng, Hanxun Bai, Hao Ren, Junhu Yao, Xiurong Xu

**Affiliations:** College of Animal Science and Technology, Northwest A&F University, Yangling 712100, China

**Keywords:** Antisecretory factor (AF), Cholecystokinin 1 receptor (CCK1R), Leucine, Proteasome, Pancreatic exocrine secretion

## Abstract

The present study aimed to investigate whether leucine affects the pancreatic exocrine by controlling the antisecretory factor (AF) and cholecystokinin receptor (CCKR) expression as well as the proteasome activity in pancreatic acinar cells of dairy calves. The pancreatic acinar cells were isolated from newborn Holstein bull calves and cultured using the Dulbecco’s modified Eagle’s medium/nutrient mixture F12 Ham’s liquid (DMEM/F12). There were six treatments of leucine dosage including 0 (control), 0.23, 0.45, 1.35, 4.05, and 12.15 mM, respectively. After culture for 3 h, the samples were collected for subsequent analysis. As the leucine concentration increased from 0 to 1.35 mM, the α-amylase activity in media decreased significantly (*P*<0.05), while further increase in leucine concentration did not show any decrease in α-amylase activity. Addition of leucine inhibited (*P*<0.05) the expression of AF and CCKR, and decreased the activity of proteasome (*P*<0.05) by 76%, 63%, 24%, 7%, and 9%, respectively. Correlation analysis results showed α-amylase secretion was negatively correlated with leucine concentration (*P*<0.01), and positively correlated with proteasome activity (*P*<0.01) and the expression of CCK1R (*P*<0.01) and AF (*P*<0.05). The biggest regression coefficient was showed between α-amylase activity and proteasome (0.7699, *P*<0.001). After inhibition of proteasome by MG-132, low dosage leucine decreased (*P*<0.05) the activity of proteasome and α-amylase, as well as the expression of CCK1R. In conclusion, we demonstrated that the high-concentration leucine induced decrease in α-amylase release was mainly by decreasing proteasome activity.

## Introduction

Pancreas releases digestive enzymes including amylase, trypsin, and lipase, which can correspondingly digest carbohydrate, protein, and fat in animal intestine. In view of the deficiency of ruminants’ intestinal starch digestibility [1], improvement in secretion of pancreatic amylase is of great significance to enhance growth, reproduction, and utilization efficiency.

In addition to being the basic unit for the synthesis of proteins, peptides, and small peptides, leucine is involved in regulation of gene expression [2], protein expression [3], cell signal pathways [4], and other physiological activities. Recent studies have shown the efficacious roles of leucine on promoting pancreatic juice secretion in dairy goat [5] and cow [6], while reduced secretion of pancreatic enzymes in rat [7]. These inconsistent results indicated that the interactions occurring *in vivo* complicate the interpretation of experimental data; while *in vitro* experiment, which uses the primary acinar cells as the model, may help us understand the molecular mechanism of secretory regulation in exocrine pancreas [8].

Antisecretory factor (AF) is a protein secreted in plasma and other tissue fluids in mammals with confirmed antisecretory function [9]. Research suggested that the direct inhibitory action of AF on pancreatic exocrine secretion in rat’s pancreatic acinar cell is related to a reduction of CCK 1 receptor (CCK1R) [10], which is a receptor of cholecystokinin (CCK). CCK is the major gastrointestinal hormone regulator of exocrine pancreatic function [11], and it promotes pancreatic exocrine secretion in pancreatic acinar cells. Leucine could stimulate the production of CCK and glucagon-like peptide-1 (GLP-1) in the gastrointestinal tract, which depended on the CCK1R expression [12]. It was reported that leucine suppressed transcription of the proteasome and muscle proteolysis in skeletal muscles [13]. But there is no information about the effect of leucine on proteasome in pancreatic acinar cells.

AF is a subunit of 26S proteasome [14]. It is unclear that whether the antisecretory function of AF is related to proteasome. In addition, another big suspense is whether the stimulatory effect of leucine on the secretion of pancreatic enzymes has any link with the AF. If leucine also had inhibitory effect on proteasome activity in pancreatic acinar cells and the AF’s antisecretory function is related to proteasome, then leucine’s suppression to proteasome probably lead to the suppression to AF with subsequent benefit to the functions of CCK by increasing CCK1R, which will then stimulate the secretion of pancreatic enzyme.

To discuss the above question, the primary purposes of the present study were as follows: (i) to examine the effect of leucine administered on pancreatic enzyme secretion in pancreatic acinar cells of dairy calves; (ii) to investigate the effect of leucine on proteasome activity and AF expression in dairy calves’ pancreatic acinar cells; (iii) to evaluate the effect of proteasome inhibitor on the activity of amylase and the expression of CCK1R in ruminant pancreatic acinar cells.

## Materials and methods

In the present study, animal experiments were approved by the Institutional Animal Care and Use Committee and were carried out strictly in compliance with the guidelines for the care and use of experimental animals at Northwest A&F University (protocol number NWAFAC1008).

### Cell isolation

Pancreatic tissues from a healthy Holstein bull calf were acquired for isolating pancreatic acinar cells. The method of cell isolation was followed the procedure of Guo et al. [15,16]. Briefly, the collected pancreatic tissue was digested in a dissociation medium containing collagenase III (1 mg/ml) in Kreb–Ringer bicarbonate (KRB) buffer with 5% BSA and incubated for 15 min with constant shaking until a homogenous solution was obtained. Then 5 ml of fresh bovine serum were added in the buffer before centrifuging it at 500×***g*** for 30 s. The cell pellet obtained was washed twice followed by centrifugation, and was then cultured in suspension or in monolayer at 37°C with 5% CO_2_.

### Cell culture and treatments

The isolated cells were seeded on 6-well plastic cell culture plates at 37°C in 5% CO_2_. Each well had 1×10^6^ cells. The Dulbecco’s modified Eagle’s medium/nutrient mixture F12 Ham’s liquid (DMEM/F12) medium (Thermo Scientific, Logan, UT, U.S.A.) was used as the basal culture medium, which was supplemented with 10% fetal bovine serum, 10 kU/l penicillin/streptomycin, 10 ng/ml epidermal growth factor, 5 μg/ml bovine insulin, and 0.25% soybean trypsin inhibitor. The amino acid concentration in each treatment was shown below: 0.05 mM l-alanine, 0.70 mM l-arginine, 0.05 mM l-aspartic acid, 0.10 mM l-cystine, 0.05 mM l-glutamic acid, 2.50 mM l-glutamine, 0.25 mM glycine, 0.15 mM l-histidine, 0.42 mM l-isoleucine, 0.45 mM l-valine, 0.50 mM l-lysine, 0.12 mM l-methionine, 0.21 mM l-phenylalanine, 0.15 mM l-proline, 0.25 mM l-serine, 0.45 mM l-threonine, 0.04 mM l-tryptophan, and 0.21 mM l-tyrosine.

There were five leucine groups which were correspondingly added with 0.23, 0.45, 1.35, 4.05, and 12.15 mM leucine. The control group was 0 mM leucine (The custom culture medium of Thermo Scientific). After incubation for 3 h, cells were harvested by scraping in the presence of ice-cold lysis buffer containing 1% (v:v) of protease and phosphatase inhibitors cocktail (Roche, Mannheim, Baden-Württemberg, Germany). Cell lysates from the 6-well plate of each medium were combined. The culture medium was also collected for subsequent analysis. The cell culturing was repeated for three times. On each time, cells from a calf were cultured in four 6-well culture plates with six kinds of media, respectively. Therefore, each group had a total of three replicates from three calves (*n*=3).

### Analysis of enzyme release in culture media

The α-amylase activity in the culture media was determined using the commercial kits (Nanjing Jiancheng Bioengineering Institute, Nanjing, China). The enzyme activity was expressed in units per liter (culture medium). One unit was defined as 1 μmol product released per minute at 39°C.

### Measurement of proteasome activity in pancreatic acinar cells

The proteasome activities were measured using the Fluorometric Proteasome 20S Assay Kit (Sigma-Aldrich, U.S.A.) according to the manufacturer’s instructions. This kit measures the chymotrypsin-like protease activity associated with the proteasome complex in cultured cells by a homogeneous fluorescent assay, and uses LLVY-R110 as a fluorogenic substrate for proteasome activities. Cleavage of LLVY-R110 by proteasome can generates strongly green fluorescent R110 (λ_ex_ = 480–500 nm/λ_ex_ = 520–530 nm). The fluorescent intensity was measured with Synergy™ HT Multi-Mode Microplate Reader (BioTek, U.S.A.).

### Protein preparation and Western blot

Protein concentration in cell lysate was determined using the Pierce bicinchoninic acid (BCA) assay kit (Thermo fisher, Rockford, IL, U.S.A.). The protein samples were boiled at 100°C for 5 min in 5× sample buffer (Beijing CoWin Biotech Co., Ltd., Beijing, China). The protein extracts (30 μg protein each) were electrophoresed in 6–15% SDS-polyacrylamide gels. The separated proteins were then transferred onto a nitrocellulose membrane (Pall Corp., Port Washington, NY, U.S.A.) in Tris–glycine buffer containing 20% methanol. The membranes were blocked and immunoblotted with a 1:1000 dilution of a primary antibody including anti-β-actin (Beijing CoWin Biotech Co., Ltd., Beijing, China, catalog number CW0096M), anti-S5a/PSMD4 (Cell Signaling Technology, Danvers, MA, U.S.A., catalog number 3846), and anti-CCK1R (Abcam, Shanghai, China, catalog number ab75153).

The proteins were detected using either goat anti-rabbit IgG (H+L)-HRP conjugated secondary antibody (1:3000) or goat anti-mouse IgG (H+L) secondary antibody (1:5000) with chemiluminescence (ECL) Western blot detection reagents (Bio-Rad, Hercules, CA, U.S.A.). β-Actin was used as the internal control. Western blots were developed and quantified using ImageJ software.

### Detection of proteasome inhibitor’s effect on amylase secretion as well as AF and CCK1R expression

The proteasome inhibitor MG-132 was purchased from Selleck (China) and dissolved in dimethyl sulfoxide (DMSO). The working concentration of MG-132 was 5 μM. There were six treatments: 0 mM leucine, 0 mM leucine + MG-132, 0.45 mM leucine, 0.45 mM leucine + MG-132, 12.15 mM leucine, 12.15 mM leucine + MG-132. Each treatment has three repeats, and the treated cells were incubated for 3 h. After incubation, cells were harvested to measure the CCK1R expression and proteasome activity, and the culture media was collected to measure the α-amylase activity.

### Statistical and analysis

The data of α-amylase activity, proteasome activity and protein expression were analyzed using one-way ANOVA model procedure and multiple comparisons of SPSS 20.0 software (SPSS Inc., Chicago, IL, U.S.A.). Pearson’s correlation analysis test and regression analysis (SPSS 20.0 software) were used to analyze the relationship between the α-amylase secretion and the proteasome activity as well as the expression of CCK1R and AF. The total protein expression levels were calculated as the ratio of the band intensity of β-actin. Significant differences were declared at *P*<0.05, and data were presented as means ± standard error of the mean (SEM).

## Results

### Effects of leucine on α-amylase secretion in culture media

**Figure 1:**
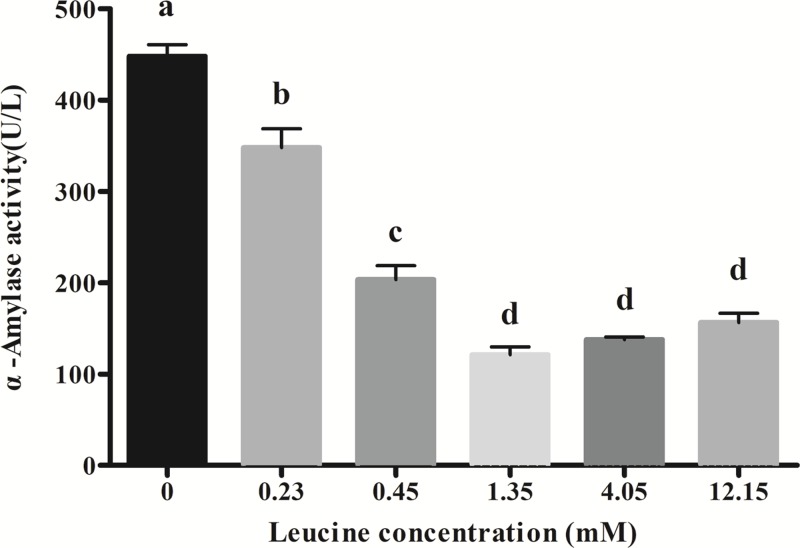
Effects of leucine on amylase secretion in calves’ pancreatic acinar cells Data are expressed as means SEM, *n*=3. Different letters mean significantly different (*P*<0.05).

Figure 1 shows effects of leucine dosages in culture media on α-amylase secretion in pancreatic acinar cells of dairy calves. As leucine concentration increased from 0 to 1.35 mM, the α-amylase release decreased significantly and reached the lowest level at 1.35 mM leucine (*P*<0.05). A further increase in the leucine concentration (4.05 and 12.15 mM) did not reduce the α-amylase release level any more (*P*>0.05).

### Effects of leucine on AF and CCK1R expression in pancreatic acinar cells

**Figure 2:**
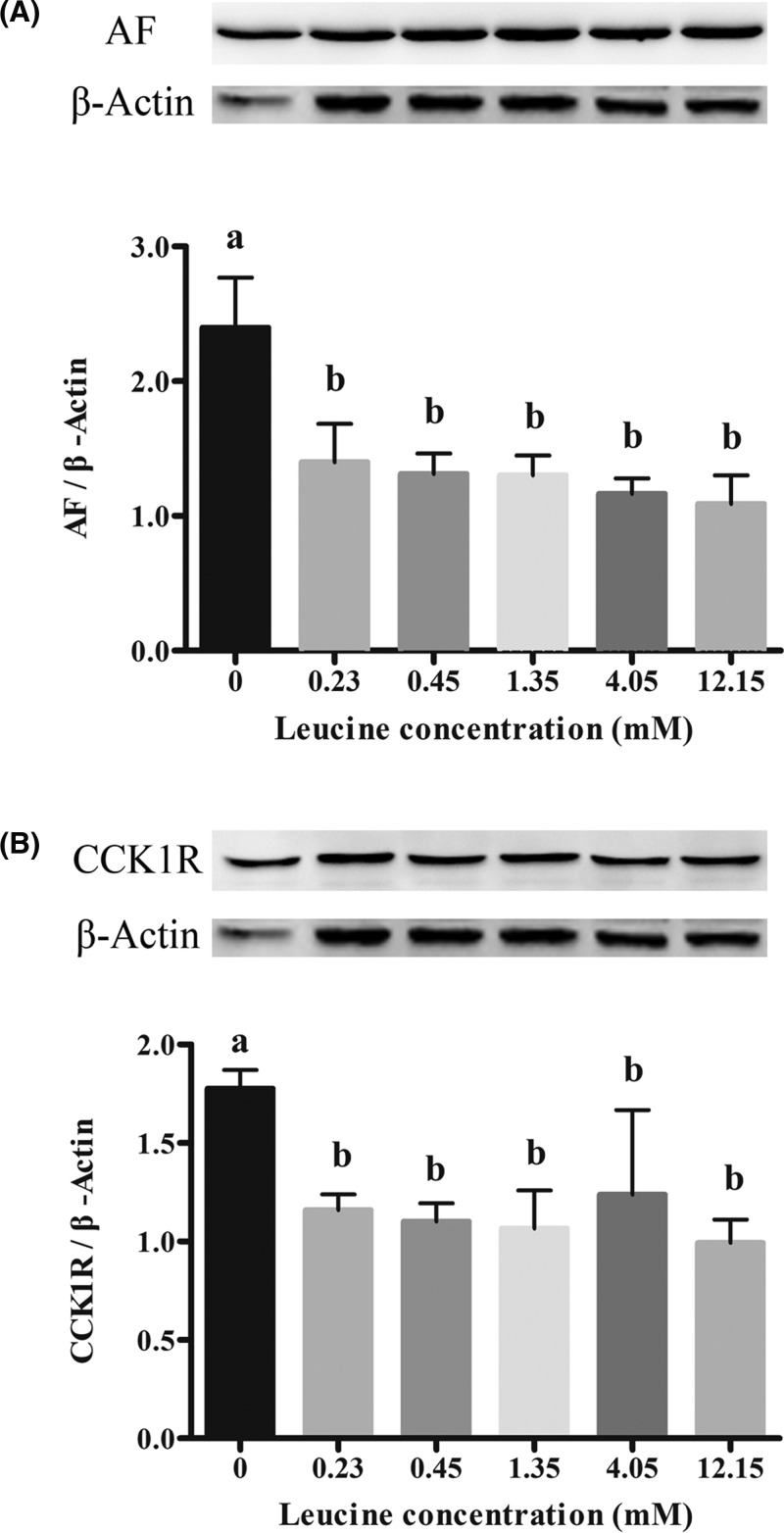
Effects of leucine on protein abundance of AF and CCK1R in calves’ pancreatic acinar cells (**A**) Represent the expression of AF. (**B**) Represent the expression of CCK1R. Data are expressed as means SEM, *n*=3. Different letters mean significantly different (*P*<0.05).

Figure 2 shows effects of leucine dosages in culture media on AF and CCK1R expression. Compared with control (0 mM leucine), leucine treatments significantly decreased the expression of AF (Figure 2A) and CCK1R (Figure 2B) (*P*<0.05). When leucine concentration increased from 0.23 to 12.15 mM, no decrease in the expression of AF and CCK1R was observed (*P*>0.05).

### Effects of leucine on proteasome activity in pancreatic acinar cells

**Figure 3:**
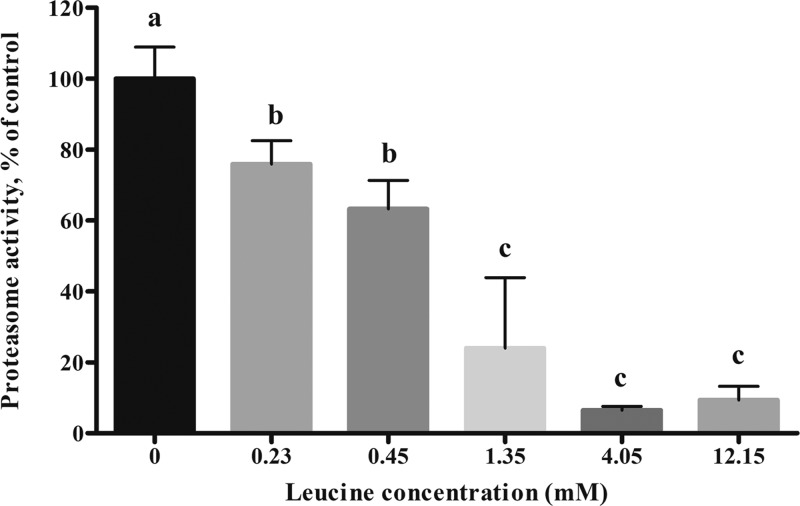
Effects of leucine on proteasome CT-like activity in calves’ pancreatic acinar cells Data are expressed as means SEM, *n*=3. Different letters mean significantly different (*P*<0.05).

Figure 3 shows effects of leucine dosages in culture media on proteasome CT-like activity. The results showed that compared with the control group (0 mmol/l), the activities of proteasome significantly decreased (*P*<0.05) in the 0.23 and 0.45 mM leucine treatments by 76% and 63%, respectively. Proteasome activity continued to decrease (*P*<0.05) with increasing leucine concentrations from 1.35 to 12.15 mM, which were 24%, 7%, and 9%, respectively.

### Regression analysis

The results (Table 1) of correlation analysis for the data from the pancreatic acinar cells showed that α-amylase secretion was negatively correlated with leucine concentration (*P*<0.01), and positively correlated with proteasome activity (*P*<0.01) and the expression of CCK1R (*P*<0.01) and AF (*P*<0.05). Leucine concentration and proteasome activity as well as the α-amylase release and proteasome activity have bigger Pearson correlation coefficients (0.925 and 0.887, respectively) than others.

**Table 1:** Pearson correlation coefficients among parameters in dairy calves pancreatic acinar cells

Variable (*N* = 18)	Leucine	Amylase	AF	CCK1R	Proteasome
Leucine	1				
Amylase	−0.889**	1			
AF	−0.653**	0.689**	1		
CCK1R	−0.527*	0.618**	0.732**	1	
Proteasome	−0.925**	0.887**	0.666**	0.425	1

* Correlation is significant at the 0.05 level (two-tailed).

** Correlation is significant at the 0.01 level (two-tailed).

**Figure 4:**
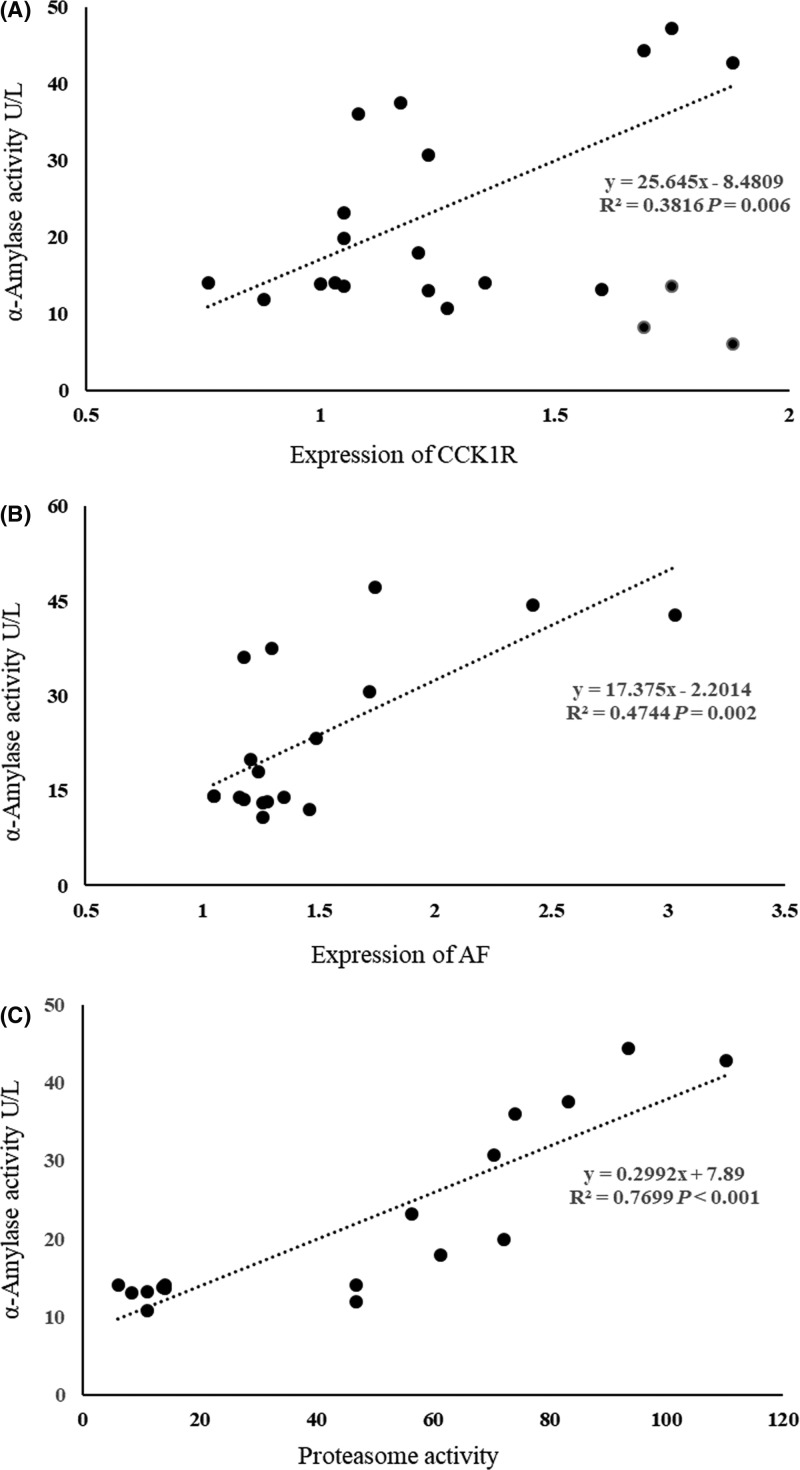
Results of regression analysis between amylase activity and the expression of CCK1R (**A**) and AF (**B**), and proteasome activity (**C**)

The results of regression analysis between α-amylase activity and CCK1R expression, AF expression and proteasome activity were presented in Figure 4. The biggest regression coefficient was shown between α-amylase activity and proteasome (0.7699, *P*<0.001). The regression models were as follows:

α-Amylase (U/l) = 25.645 × CCK1R – 8.4809 (*N* = 18; *R*^2^ = 0.3816; *P*<0.05)

α-Amylase (U/l) = 17.375 × AF – 2.2014 (*N* = 18; *R*^2^ = 0.4744; *P*<0.05)

α-Amylase (U/l) = 0.2992 × proteasome + 7.89 (*N* = 18; *R*^2^ = 0.7699; *P*<0.05)

### Effects of proteasome inhibitor (MG-132) on α-amylase secretion as well as AF and CCK1R expression

**Figure 5:**
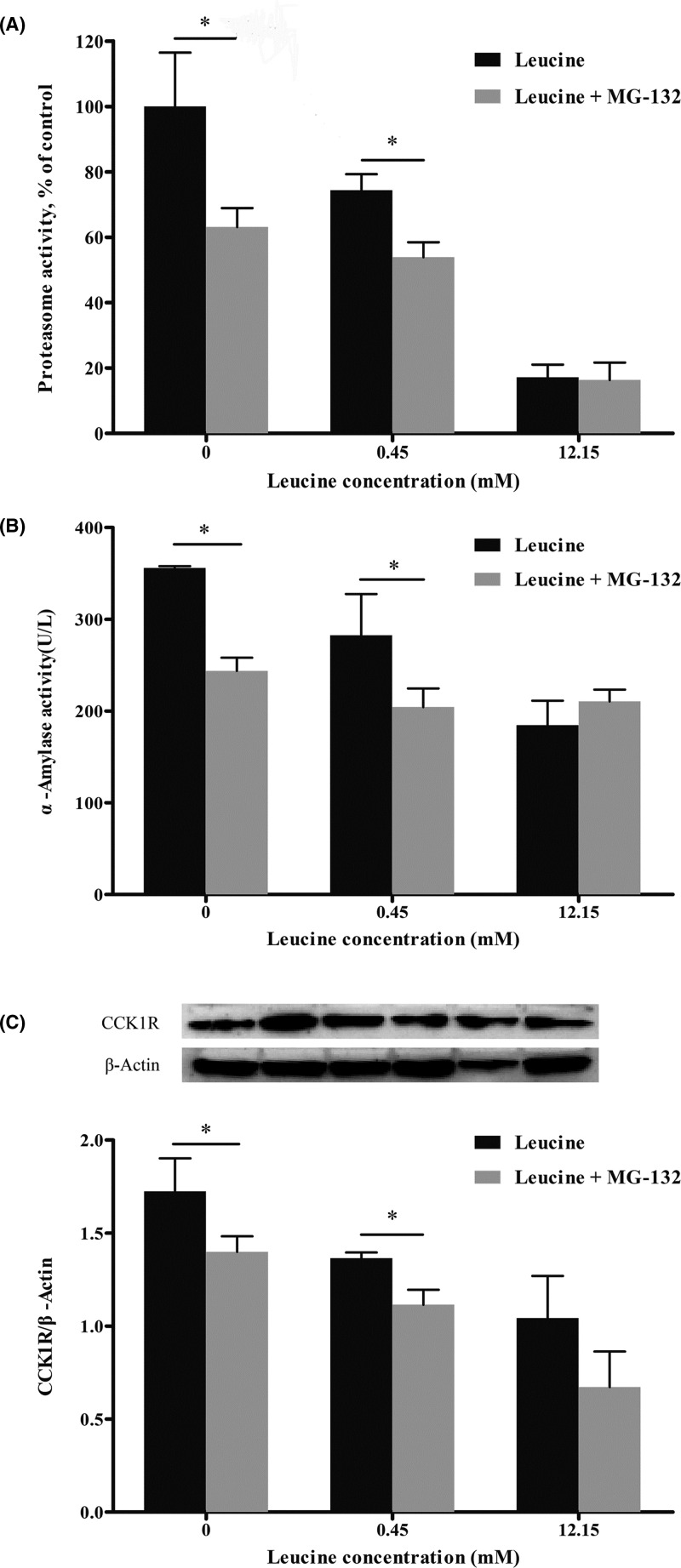
Effects of MG-132 on amylase, proteasome activity, and CCK1R expression in calves’ pancreatic acinar cells Data are expressed as means SEM, *n*=3. *Means significantly different between two groups (*P*<0.05).

Figure 5 represented the effects of MG-132 on proteasome activity, α-amylase release, and CCK1R expression. MG-132 significantly decreased (*P*<0.05) proteasome activity (Figure 5A), α-amylase release (Figure 5B), and CCK1R expression (Figure 5C) in 0 and 0.45 mM leucine treatments. But in the 12.15 mM leucine culture, supplementation with MG-132 did not affect (*P*>0.05) α-amylase release, CCK1R expression, and proteasome activity.

## Discussion

Leucine not only serves as substrates for protein synthesis but also participates in regulation of some physiological processes. In isolated perfused rat pancreas, leucine infusion could inhibit the secretion of pancreatic enzymes [7]. However, it was reported that duodenum infusion of leucine could increase secretion of pancreatic α-amylase in dairy goat [5] and Holstein cow [6]. The above studies showed that leucine could regulate the secretion of pancreatic enzymes. In the present study, fresh primary pancreatic acinar cells were used to study effects of leucine dosages on amylase release. The results obtained here shows that leucine inhibits release of α-amylase, which differs from results observed in *in vivo* ruminant experiments, but similar with the results investigated in isolated perfused rat pancreas [7]. Leucine inhibited the α-amylase release in primary pancreatic acinar cells, and when concentration of leucine increased from 0 to 1.35 mM, the α-amylase release was decreased by 73%. The differences probably derived from the difference in amino acids concentration between *in vivo* and *in vitro* experiment. Another possible reason was that the high concentration of leucine could affect the expression of other amino acids transporter [17] and the metabolism of other amino acids such as isoleucine [16]. These disorders may lead to inhibition of amylase secretion.

It was reported that leucine alone stimulated protein synthesis, and inhibited the protein breakdown, probably by reducing the activation of the ubiquitin-proteasome system [18]. As a subunit of proteasome, AF inhibited human, pig, and other animals’ intestinal fluid secretion and the pancreatic secretion in rats [9,10]. In the present study, expression of AF was decreased as leucine concentration increased, but the α-amylase release did not increase after expression of AF was inhibited. These demonstrated that AF possibly has no inhibitory effect on the exocrine function of pancreatic acinar cells of dairy calves, or the effect of AF reduction was masked by other factors, such as CCK, which may be more effective on regulating pancreatic secretion process than AF. Therefore, further research should focus on the relationship between leucine and CCK receptors or proteasome.

The results of the correlation analysis showed that α-amylase release was positively correlated with proteasome activity, but negatively correlated with leucine concentration, which indicated that α-amylase secretion depends on proteasome activity. Additionally, regression analysis results also showed a similar trend. These results suggested that procedure aiming to control the α-amylase release should regulate proteasome activity firstly.

Proteasome is an important intracellular protein complex, mainly responsible for the degradation of intracellular proteins [19]. Decrease in proteasome activity can lead to a decrease in the available amino acids substrate and a direct effect on the rate of enzyme synthesis, which may affect pancreatic secretion function. For instance, insulin secretion was inhibited when proteasome activity decreased in β-cells of rat pancreas [20]. Another mechanism of proteasome’s effect on insulin secretion is believed that proteasome inhibits the voltage-dependent calcium channels in cell membrane and then reduces intracellular Ca^2+^ concentration [21]. Leucine suppressed transcription of the proteasome and degradation of myofibrillar protein in chick skeletal muscles [22]. Busquets et al. [13] reported that leucine (10 mM solution) inhibited muscle proteolysis in the extensor digitalis and soleus muscles after incubation with a solution to activate the proteolytic system. In the present study, leucine inhibited proteasome activity, and this inhibition may lead to the decrease in secretion of digestive enzymes. Indeed, we observed similar phenomenon by inhibiting proteasome activity by using the proteasome inhibitor MG-132. Our results showed that leucine and MG-132 have synergistic function and both of them could inhibit the proteasome activity, α-amylase release and CCK1R expression. It was noteworthy that when the proteasome was inhibited by MG-132, the expression of CCK1R was also decreased. These results indicated that leucine decreases the expression of CCK1R probably by inhibiting proteasome activity. The ubiquitin-proteasome system was deemed to participate in internalization and degradation of several membrane proteins, such as epithelial sodium channels [23], growth hormone receptors [24], epidermal growth factor receptors [25], and certain immune system receptors [26].

It was reported that amino acids could inhibit pancreatic enzyme secretion by reducing Ca^2+^ concentration in pancreatic acinar cells [7]. Intracellular Ca^2+^ concentration is a direct signal for secretion of digestive enzymes. When cells were stimulated by acetylcholine (ACH) and CCK, the corresponding G protein-coupled receptors on the membrane produce the second messengers nicotinic acid adenine dinucleotide phosphate (NAADP) and inositol 1,4,5-trisphosphate (IP_3_), which can be recognized correspondingly by IP3 receptor (IP_3_R) and ryanodine receptors (RyRs) on the endoplasmic reticulum, and lead to endoplasmic reticulum Ca^2+^ release, and thus stimulates the secretion of zymogen granules [27]. Research showed that tryptophan inhibited the secretion of pancreatic amylase in a concentration-dependent manner by regulating intracellular Ca^2+^ [28]. Our study indicated that the mechanism of leucine’s inhibitory function on α-amylase release was possibly associated with the decreased CCK1R expression in pancreatic acinar cell membrane. We believe, there were two possibilities to the results we obtained in the present study, first, the CCK1R on the acinar cell membrane activates the phosphatidylinositol signaling pathway by binding to CCK and induces an increase in intracellular Ca^2+^ that mediates the secretion of zymogen granules [29] and might be the primary pathway for regulation of pancreatic exocrine function. Second, the contrary results which compared with the gastrointestinal findings [12] may be related to the different cell types of pancreas and gastrointestinal cell types.

## Conclusions

Our findings demonstrate that high-concentration leucine could decrease α-amylase release in primary pancreatic acinar cell through inhibiting proteasome activity. Our results suggested that proteasome is a possible key factor for controlling the release of digestive enzymes, and therefore indicated that it is important to focus on regulating proteasome activity in order to increase secretion of pancreatic enzymes.

## Abbreviations

AF: antisecretory factor
CCK: cholecystokinin
CCK1R: CCK 1 receptor
IP_3_: inositol 1,4,5-trisphosphate
SEM: standard error of the mean
